# Multimodal Spectroscopic Imaging of Pea Root Nodules to Assess the Nitrogen Fixation in the Presence of Biofertilizer Based on Nod-Factors

**DOI:** 10.3390/ijms222312991

**Published:** 2021-11-30

**Authors:** Katarzyna Susniak, Mikolaj Krysa, Dominika Kidaj, Monika Szymanska-Chargot, Iwona Komaniecka, Katarzyna Zamlynska, Adam Choma, Jerzy Wielbo, Leopold L. Ilag, Anna Sroka-Bartnicka

**Affiliations:** 1 Department of Genetics and Microbiology, Institute of Biological Sciences, Maria Curie-Sklodowska University, Akademicka 19, 20-033 Lublin, Poland; kasiasusniak@gmail.com (K.S.); dominika.kidaj@mail.umcs.pl (D.K.); ikoma@hektor.umcs.lublin.pl (I.K.); katarzyna.zamlynska@mail.umcs.pl (K.Z.); adam.choma@mail.umcs.pl (A.C.); jerzy.wielbo@poczta.umcs.lublin.pl (J.W.); 2 Independent Unit of Spectroscopy and Chemical Imaging, Medical University of Lublin, Chodzki 4a, 20-093 Lublin, Poland; mikolajkrysa@umlub.pl (M.K.); leopold.ilag@mmk.su.se (L.L.I.); 3 Institute of Agrophysics, Polish Academy of Sciences, Doswiadczalna 4, 20-290 Lublin, Poland; m.szymanska@ipan.lublin.pl; 4 Department of Materials and Environmental Chemistry, Stockholm Univeristy, Svante Arrhenius Väg 16 C, 106-91 Stockholm, Sweden

**Keywords:** *Rhizobium*, Nod factor, pea, symbiosis, MALDI MSI, Raman spectroscopy, FT-IR spectroscopy

## Abstract

Multimodal spectroscopic imaging methods such as Matrix Assisted Laser Desorption/Ionization Mass Spectrometry Imaging (MALDI MSI), Fourier Transform Infrared spectroscopy (FT-IR) and Raman spectroscopy were used to monitor the changes in distribution and to determine semi quantitatively selected metabolites involved in nitrogen fixation in pea root nodules. These approaches were used to evaluate the effectiveness of nitrogen fixation by pea plants treated with biofertilizer preparations containing Nod factors. To assess the effectiveness of biofertilizer, the fresh and dry masses of plants were determined. The biofertilizer was shown to be effective in enhancing the growth of the pea plants. In case of metabolic changes, the biofertilizer caused a change in the apparent distribution of the leghaemoglobin from the edges of the nodule to its centre (the active zone of nodule). Moreover, the enhanced nitrogen fixation and presumably the accelerated maturation form of the nodules were observed with the use of a biofertilizer.

## 1. Introduction

Legume plants are able to enter into a symbiotic relationship with soil bacteria belonging to the rhizobia group; the legume plants provide a source of nitrogen for the soil bacteria, while the bacteria provide a source of carbon for the plants. As a result of this symbiotic relationship, specific organs called nodules form on the plant roots, and occasionally appear on stems. This is a unique process, where rhizobia, after converting their cells into nitrogen-fixing bacteroids, can reduce nitrogen gas in ammonia, producing, amino acids, including glutamate and asparagine, among others [1]. Generally, rhizobia are only able to induce the development of root nodules on a specific plant-host, and the morphology of the nodules depends on the geographic origin of the plant, which varies according to the legume clade [2]. Two basic types of nodules with different morphologies are usually described: indeterminate nodules (e.g., formed at pea) possessing a persistently growing tip with active meristem, and determinate nodules (e.g., formed at soya), without meristematic cells in a mature form. The nitrogen fixation process occurs in the central part of a nodule, where bacteroids are concentrated [2].

The mutual recognition of symbiotic partners is based on the exchange of specific molecular signals originating from plants and bacteria. Plant roots secrete a group of cyclic compounds–flavonoids, whereas rhizobia, in response, produces lipochitooligosaccharides (so called Nod factors) [3,4,5]. Nod factors are key signal molecules that initiate symbiotic interactions and are essential for establishing effective nitrogen-fixing symbiosis between rhizobia and legume plants [6]. Nod factors trigger the process of nodule initiation and are involved in the formation of the root nodule through the regulation of auxin levels (primarily indole-3-acetic acid (IAA)) in plants [7]. It has been proven that Nod factors also have an impact on many processes in plants, such as the stimulation of seed germination, promotion of root branching or shoot growth, and others unrelated to root nodule development [8]. Preparations containing Nod factors (biofertilizers) stimulate the increase of the legume and non-legume (e.g., cotton, maize) plant biomass [9,10,11,12,13]. Seed exposure to Nod factors before sowing (e.g., solution soaking) accelerates the process of establishing symbiosis and increases the number of root nodules that can be colonized by indigenous rhizobia present in soil [9,11,12]. The application of isolated Nod factors results in an increase in atmospheric nitrogen fixation, even at low symbiotic efficiency of indigenous soil strains, infecting plant root nodules.

In the central part of the mature and functional nodule, where the nitrogen fixation process occurs, a high concentration of leghaemoglobin is found. Leghaemoglobin binds to O_2_, minimizing its level to protect the oxidation of the specific enzyme that reduce N_2_ to ammonia, nitrogenase. The globin part of leghaemoglobin is produced by the plant in response to bacterial infection, but the second part—the heme—is synthesized by the rhizobia cells [14].

Advanced analytical techniques: mass spectrometry imaging (MSI) and FT-IR as well as Raman spectroscopic imaging were used in this study. MSI is a powerful tool for label-free visualization of the spatial distribution of plant metabolites directly in the tissue. This technique allows for identification of primary and secondary metabolites such as proteins, peptides, amino acids, lipids, flavonoids, and organic acids [15,16]. FT-IR and Raman spectroscopy are vibrational methods widely used in the chemical analysis of plant tissues. Both techniques allow structural characterization of plant cell wall polysaccharides, plant pigments, proteins, phenolics and lipids [17,18]. In particular, Raman and FTIR spectroscopy combined with microscopic techniques gives the possibility to visualize changes in physicochemical properties of cell wall polysaccharides during plant development or fruit senescence [19,20,21], or during pathogen attack [22]. Taken together, all techniques can be applied to study changes in nodule composition, morphology as well as the characteristics and localization of substances which are induced after application of the fertilizers.

The aim of this work was to assess the influence of newly prepared biofertilizer containing Nod factors on plant growth by monitoring changes in the distribution of selected metabolites related to nitrogen fixation in root nodules. Analytes of interest included: auxins, flavonoids, endogenous amino acids, leghaemoglobin, and proteins and lipids in general. The influence of two biofertilizer preparations were examined: (i) crude concentrated butanolic extract of spent medium, and (ii) fraction of the extract containing an increased content of Nod factors.

## 2. Results and Discussion

### 2.1. Isolation and Purification of the Biofertilizer Containing Nod Factors

**Figure 1:**
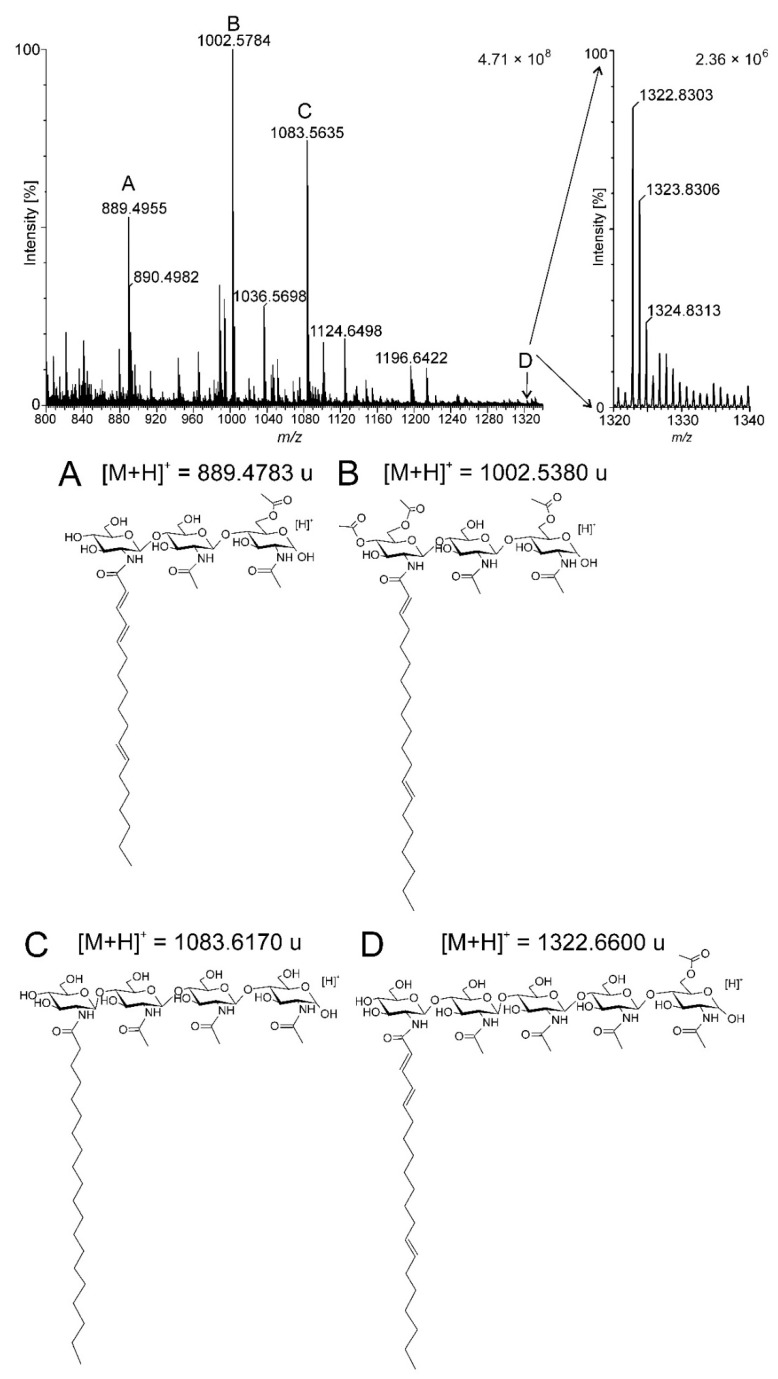
ESI-MS spectrum of purified biofertilizer (fraction eluted with 60% acetonitrile) in positive polarity. (**A**–**D**)—chemical formulas of Nod factors identified.

Fraction of the preparation containing Nod factors eluted with 60% acetonitrile was analysed with UPLC-ESI MS. The TUV detector connected to the UPLC instrument allowed observation of chromatographic peaks at a retention time of about 10–13 min. The mass spectrum for this region showed a series of signals that could be assigned based on the literature data [23], corresponding to the Nod factors molecules produced by *R. leguminosarum* (Figure 1 and Table 1). It can be assumed, based on high-resolution mass spectrometry analysis, that the lipochitooligosaccharide molecules present in the preparation consist of three to five glucosamine residues (GlcN), one fatty acid molecule and from one to three *O*-acetyl groups (Ac). The most frequent were fractions of lipochitooligosaccharide composed of three and four glucosamine units with a different degree of *O*-acetylation (Figure 1A–C). Nod-factor composed of five glucosamine units constituted minor fraction of the biofertilizer (Figure 1D).

### 2.2. Influence of the Biofertilizer on the Plant Development

**Figure 2:**
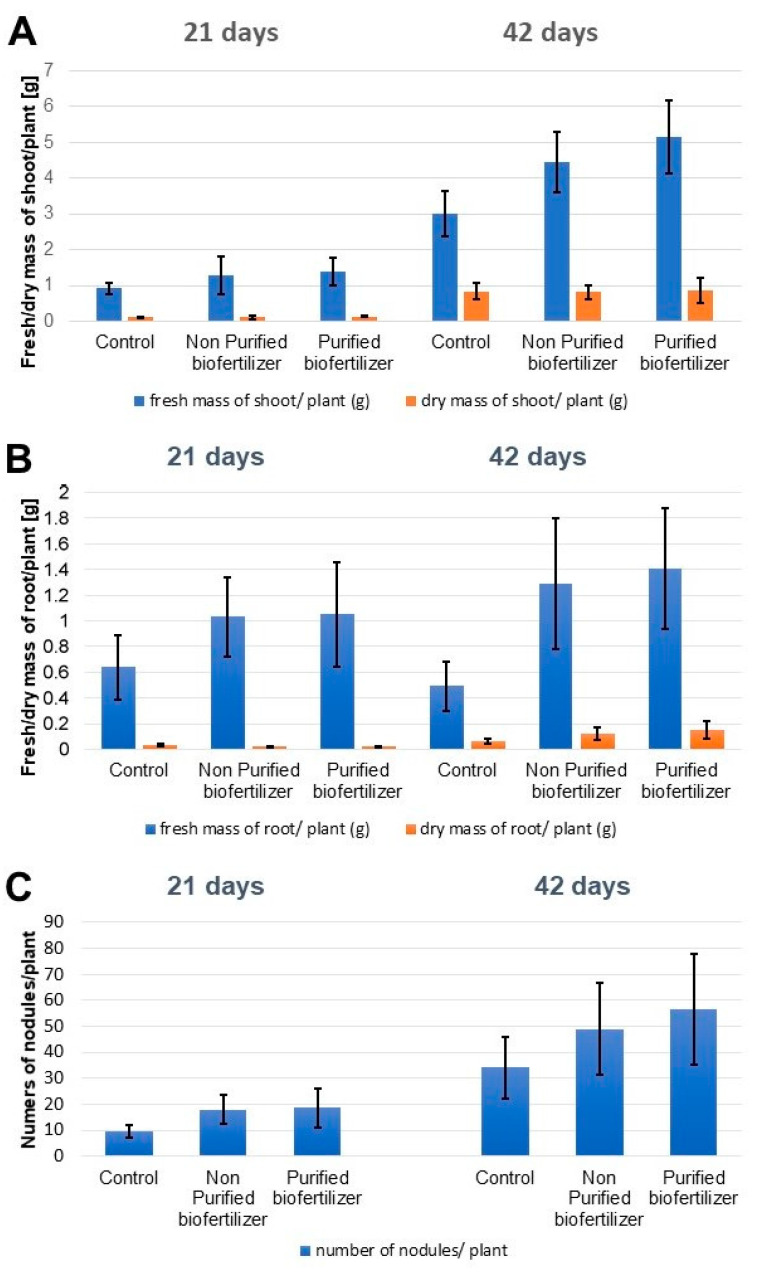
Effect of purified and non-purified biofertilizer on the growth of pea plants (*Pisum sativum* L. cv. Batuta) in greenhouse conditions. (**A**) fresh and dry mass of the shoots, (**B**) fresh and dry mass of the roots (**C**) the number of root nodules.

The influence of biofertilizers on the plant development of fresh and dry mass is presented in Figure 2. The obtained results indicate that supplementation of plants (seeds) with the Nod factors-containing biofertilizers has a beneficial effect on plant growth. The strongest differences in fresh and dry mass of plants’ shoots and roots were visible after 42 days of growth. Treatment of seeds with purified biofertilizer improved the shoots growth twice compared to the control (Figure 2A). The fresh and dry mass of roots from plants treated with non-purified and purified biofertilizer increased more than twice compared to the control (Figure 2B). This trend is also visible in the growth of younger plants (after 21 days of growth) The increased number of nodules per plant correlates with increased fresh and dry mass of shoots and roots of plants treated with biofertilizers in comparison to the control (Figure 2C).

### 2.3. MALDI MSI

**Figure 3:**
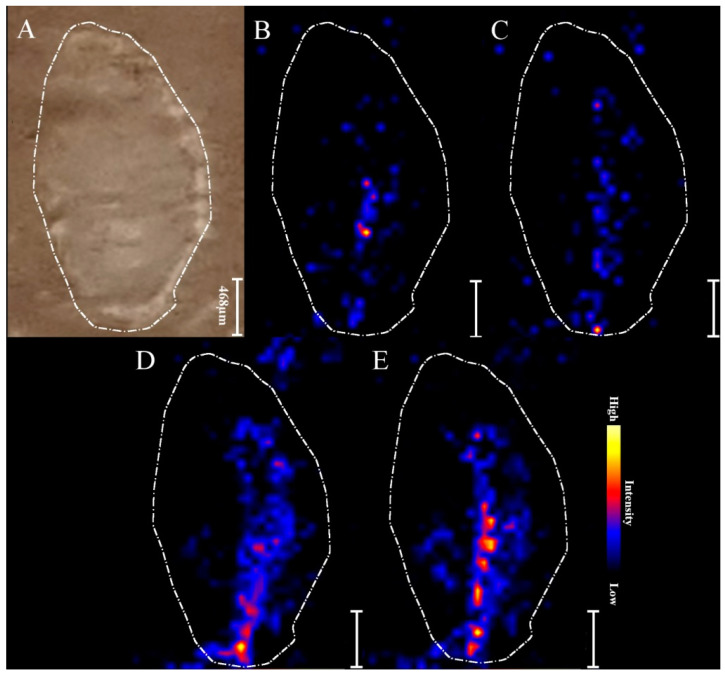
MALDI MSI images of distribution of the metabolites within root nodule: (**A**) Photograph of a root nodule section on a glass slide covered with DHB matrix and chemical maps of: (**B**) asparagine (133.06 *m*/*z*), (**C**) indole-3-butyric acid (204.1 *m*/*z*), (**D**) hesperetin (303.02 *m*/*z*), and (**E**) leghaemoglobin (617.17 *m*/*z*) of 21-day root nodule from plant grown from seeds coated with the non-purified biofertilizer.

MALDI MSI was performed on root nodules of *Pisum sativum* longitudinal sections in order to identify and visualize metabolites related to biological nitrogen fixation. Four distinctive plant metabolites were selected to investigate changes in their distribution in root nodules in order to assess the effectiveness of biofertilizers in the biological nitrogen fixation. Figure 3 illustrates the MS images of selected metabolites in a root nodule under the influence of non-purified biofertilizer. Figure 3A shows a photograph of a nodule section with applied matrix. Figure 3B–E represents distribution of asparagine (133.06 *m*/*z*), indole-3-butyric acid (204.1 *m*/*z*), hesperetin (303.02 *m*/*z*) and heme (617.17 *m*/*z*), respectively (as described in [15]).

The concentration of selected metabolites differs throughout the nodule as they concentrate mostly in the central part of nodule, as expected with respect to its morphology. Ions corresponding to heme from leghaemoglobin were selected for preparing the MALDI MS images [15].

**Figure 4:**
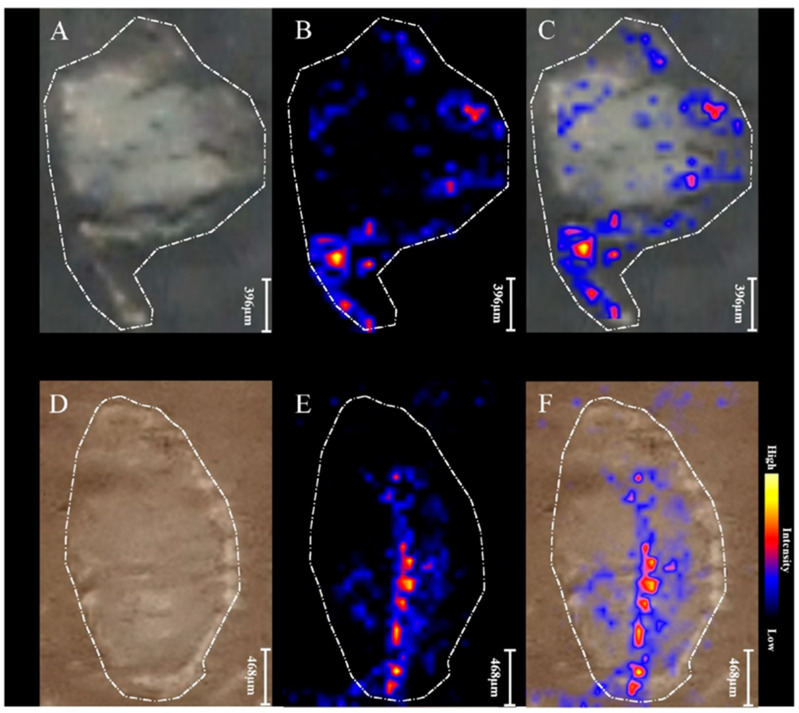
MALDI MSI images of 21-day old root nodules from plants grown from seeds coated with non-purified biofertilizer (**D**–**F**) vs. 21-day old root nodules from control sample (**A**–**C**). (**A**,**D**) Photographs of a root nodule sections on a glass slides covered with DHB matrix; (**B**,**E**) MALDI-MSI chemical maps of leghaemoglobin (617.17 *m*/*z*); (**C**,**F**) overlapped all individual chemical images.

The spatial distribution of heme (shown in Figure 4) in the 21 days-old nodule from plant grown from seeds treated with non-purified biofertilizer differs to the control. In the nodules after biofertilizer application the signals on chemical maps were observed in the central part of section, while in the control, the signals were located mainly on the edges.

**Figure 5:**
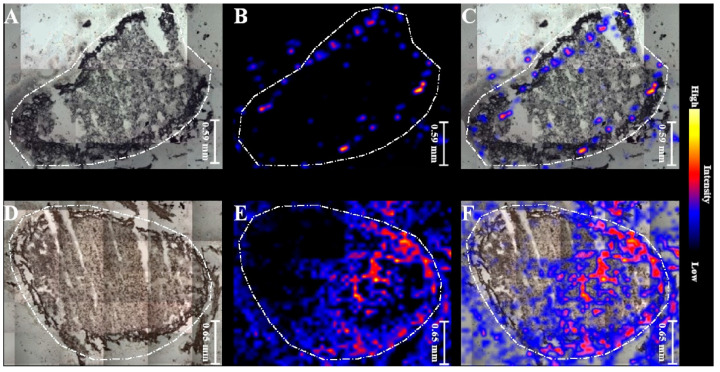
MALDI MSI images of root nodules from 42-day old plant: (**A**–**C**) control samples and (**D**–**F**) root nodule from plant grown from seeds coated with purified biofertilizer. (**A**,**D**) Light microscopic images; (**B**,**E**) MALDI-MSI chemical maps of leghaemoglobin (617.17 *m*/*z*); (**C**,**F**) overlapped chemical images and light microscopic images.

After 42 days of plant growth, fully developed and mature nodules were collected and subjected to MSI analysis (Figure 5). In the control samples the heme from leghaemoglobin was spaced on the edges in contrast to nodules derived from plants grown from seeds treated with purified biofertilizer, where heme was situated in the centre of nodule, which is biologically the most efficiently functioning part of the organ [2]. The differences in spatial distribution of leghaemoglobin in nodules indicated that the presence of Nod factors had an influence on nodules development and maturation to more productive forms. MALDI MSI chemical maps are convergent with results obtained in greenhouse tests.

### 2.4. Infrared Spectra with Statistical Analysis

**Figure 6:**
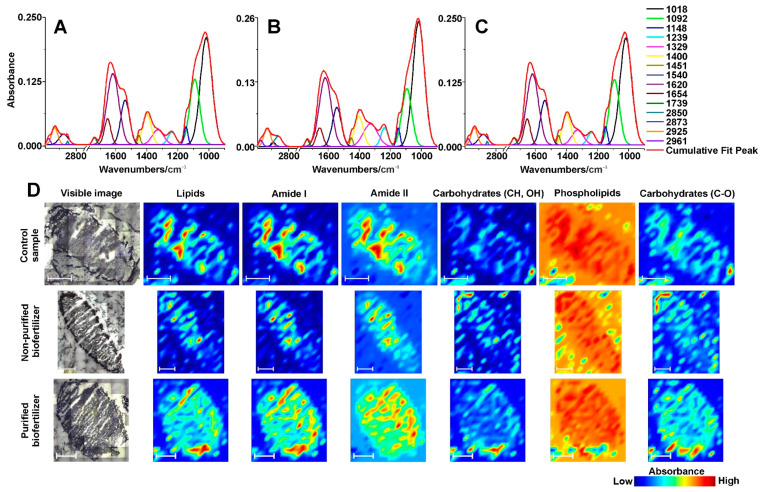
FT-IR deconvoluted spectra and chemical maps of the root nodules. (**A**) deconvoluted, average spectrum of the control sample; (**B**) deconvoluted, average spectrum of the nodule from plant grown from seeds coated with non-purified biofertilizer; (**C**) deconvoluted, average spectrum of the nodule from plant grown from seeds coated with purified biofertilizer; (**D**) chemical maps presenting the distribution of the selected bands on the root nodules. Each line of the spectra contributes to different band. The white scale bar is 750 µm.

To perform a more detailed interpretation of IR spectra, statistical analysis was carried out. The aim of this analysis was to monitor the chemical differences between the samples. The IR spectra of three set of samples (after the influence of purified and non-purified biofertilizers, as well as control samples) were averaged and deconvoluted (Figure 6A–C). In all of the spectra from the root nodules (Figure 6A–C) the same bands were detected. The band at 1018 cm^−1^, 1092 cm^−1^ and 1148 cm^−1^ corresponded to C-O ν of carbohydrates [24]. The band at 1239 cm^−1^ corresponded to PO^2−^ ν_as_ of phospholipids [24]. The bands at 1329 cm^−1^, 1400 cm^−1^ and 1451 cm^−1^ corresponded to CH and OH σ and ρ of carbohydrates [24]. Band at 1540 cm^−1^ corresponded to Amide II (~60% NH bending vibration, ~40% CN ν) [24]. Amide I bands are sensitive to the changes of the protein secondary structure, thus the relation of the intensity of the bands within the Amide I can give an insight into the protein structure. In the nodules averaged spectra, the bands corresponded to β-sheet and α-helix structures were detected at 1620 cm^−1^ and 1654 cm^−1^, respectively [25]. The band at 1739 cm^−1^ can be ascribed to the C=O ν of lipids ([24]). Infrared spectra can also give an insight into the average length of the lipids by taking into account the ratio of the CH_2_ to CH_3_ groups of the same symmetry lipid bands. In all of the nodules average spectra, the bands corresponding to CH_2_ ν_s_ (at 2850 cm^−1^), CH_3_ ν_s_ (at 2873 cm^−1^), CH_2_ ν_as_ (at 2925 cm^−1^), CH_3_ ν_as_ (at 2961 cm^−1^) [24] could be detected.

The statistical analysis of the area under the bands showed statistically significant differences (*p* ≤ 0.05) between the control group (*n* = 8), non-purified biofertilizer group (*n* = 8) and the purified biofertilizer group (*n* = 8) in the bands corresponding to PO^2−^ ν_as_ of phospholipids (1240 cm^−1^), CH and OH σ and ρ of carbohydrates (1329 cm^−1^, 1400 cm^−1^), Amide II (1540 cm^−1^), α-helix band of Amide I (1654 cm^−1^), CH_2_ ν_as_ of lipids (at 2925 cm^−1^) and CH_3_ ν_as_ of lipids (at 2961 cm^−1^).

The HSD Tukey test of the band area at 1240 cm^−1^ ascribed to the PO^2−^ ν_as_ of phospholipids separated the nodules into two statistical groups. One group with the purified biofertilizer (average area = 1.52, SD = 0.82) and the second group with the control nodules and non-purified biofertilizer (average area = 3.02, SD = 0.49; average area = 3.08, SD = 0.41, respectively). The mature nodules have a lower amount of phospholipids than the immature ones [26]. It can be therefore concluded that the nodules from plants treated with purified biofertilizer mature faster than those from the control samples or treated with non-purified biofertilizer.

Two bands corresponding to CH and OH σ and ρ of carbohydrates at 1329 cm^−1^ and 1415 cm^−1^ also showed significant differences in the area under bands. The bands at 1329 cm^−1^ were separated into two statistical groups, one with the purified biofertilizer group and control group (average area = 4.02, SD = 1.09; average area = 4.39, SD = 0.97, respectively), second with non-purified biofertilizer (average area = 6.89, SD = 1.25). The bands at 1400 cm^−1^ were also separated into two statistical groups, one with purified biofertilizer and the control group (average area = 4.12, SD = 0.67; average area = 4.75, SD = 0.78, respectively) and second with the control (as before) and non-purified biofertilizer group (average area = 5.67, SD = 0.88). The control group was assigned to both statistical groups. This means that the significant difference is between the purified and non-purified group. Despite significant differences in the area of the given bands this carbohydrate region is not specific to particular carbohydrates. Moreover, these are not the only carbohydrate bands present in these samples, therefore it cannot be specified what changes occurred in terms of carbohydrates in the nodules; however, these results suggest that the amount or species of carbohydrates changed.

The area under the bands at 1540 cm^−1^ assigned to the Amide II also presented significant differences and divided the spectra of nodules into two statistical groups. First one containing the control and non-purified biofertilizer group (average area = 7.24, SD = 1.01; average area = 8.54, SD = 0.98), second with non-purified biofertilizer group (as before) and purified biofertilizer (average area = 8.85, SD = 1.53). The influence of the proteins structure on the shape of Amide II band is complex, thus these changes cannot be characterized in the biological sample [27].

Area under the bands, corresponding to α-helical structures of Amide I (at 1656 cm^−1^), was also significantly different between the samples. The spectra were separated into two statistical groups, one containing the control and non-purified biofertilizer group (average area = 0.51, SD = 0.27; average area = 0.53, SD = 0.48, respectively) and second group with purified biofertilizer (average area = 1.42, SD = 0.83). Moreover, the ratio of the α-helix to β-sheet structures of the proteins were calculated (band area at 1654 cm^−1^ to band area at 1620 cm^−1^ [25]). The highest amount of α-helical proteins was found in the purified biofertilizer group, moreover it presented the highest percentage of the α-helical to β-sheet structures of the proteins (ratio = 0.13109). The case of non-purified biofertilizer group is different, because the amount of the produced α-helical proteins, as well as the ratio of the α-helix to β-sheets protein structure (ratio = 0.04206) was almost at the same level as in the control group (ratio = 0.04207). One of the most abundant proteins produced by the nodules is leghaemoglobin. According to Escuredo and co-workers [28] leghaemoglobin accounts for over 21% of the soluble proteins in the root nodules. It provides a micro-anaerobic environment inside the nodule, which is essential for the proper nitrogen fixation [29]. Since leghaemoglobin is a mostly α-helical protein, it can be suggested that the high amount α-helical proteins in the nodules from plants treated with purified biofertilizer is linked with a high amount of leghaemoglobin.

Two bands of the CH_2_ ν_as_ at 2925 cm^−1^ and CH_3_ ν_as_ at 2961 cm^−1^ of the lipids were also significantly under the influence of the biofertilizers. The band corresponding to CH_2_ ν_as_ was separated into two statistical groups, first with purified biofertilizer and control group (average area = 1.69, SD = 0.32; average area = 1.84, SD = 0.21, respectively), second with control (as before) and non-purified biofertilizer group (average area = 2.06, SD = 0.27). The band corresponded to CH_3_ ν_as_ was also divided into two statistical groups, one with non-purified biofertilizer and control group (average area = 0.27, SD = 0.07; average area = 0.31, SD = 0.07, respectively), second with purified biofertilizer group (average area = 0.42, SD = 0.11). The ratio of these two bands provides the information about the changes of the lipid’s length. The longest lipid chains were found in the non-purified biofertilizer (ratio = 8.33), shorter were in the control (ratio = 6.07) and the shortest were found in purified biofertilizer group (ratio = 4.31). These results suggest that non-purified biofertilizer induce the elongation of the lipids and purified biofertilizer induces abridgement of the lipids in the root nodules.

### 2.5. FT-IR Spectroscopic Imaging

In order to determine the distribution of the selected metabolites in the root nodules affected by the use of biofertilizers the chemical maps were obtained (Figure 6D). The bands visualized were chosen in respect to the main spectra region: lipids, Amide I, Amide II, numerous bands of carbohydrates, phospholipids, and C-O bands of carbohydrates.

In the control, nodule lipids (3002–2808 cm^−1^), Amide I (1707–1579 cm^−1^) and Amide II (1579–1480 cm^−1^) are distributed similarly—these bands are mainly on the edges of the nodule. Both of the bands corresponded to carbohydrates (1460–1270 cm^−1^ and 1087–837 cm^−1^) and the band corresponding to phospholipids (1270–1182 cm^−1^) are distributed evenly throughout the sample.

The chemical map of nodule from plants grown from seeds treated with non-purified biofertilizer presents that the lipids, Amide I and Amide II bands are distributed mainly in the centre of the sample—the region responsible for the nitrogen fixation. The carbohydrates are distributed mainly on the edges of the sample, but small amounts of it could be found in the centre of the nodule. The phospholipid band is distributed evenly throughout the sample (Figure 6D).

The FT-IR image of the nodule from plants grown from seeds treated with purified biofertilizer shows that the lipid band is distributed evenly throughout the nodule, but in-depth analysis shows that slightly more lipids are at the edges of the sample. The band corresponding to Amide I and Amide II is distributed mainly in the centre of the nodule, however a large amount of it could be also found on the edges. The numerous bands of carbohydrate region are mainly distributed on the edges of the sample. The carbohydrate region corresponding to C-O group was distributed almost evenly throughout the sample, however the highest concentration is on the edges of the nodule. In the case of phospholipid bands, they are distributed evenly throughout the sample.

The highest differences among the control and the samples obtained using nodules from plants grown from seeds treated with both biofertilizers were found in the distribution of Amide I and Amide II regions. In the control sample these bands were distributed mainly on the edges of the sample, while in the experiments with biofertilizers they were located in the centre of the sample. These results suggest, that the leghaemoglobin, which is crucial for nitrogen fixation, is mainly located in the centre of the sample from plants grown from seeds coated with biofertilizers. However, the nodules from plants grown from seeds treated with purified biofertilizer present the leghaemoglobin in the centre of the root nodule with higher concentration than in nodules from the plant with seeds coated with the non-purified biofertilizer. The centre zone of the nodules are considered to be responsible for the nitrogen fixation and these results confirm this statement. It is also consistent with the mass spectrometry imaging results.

### 2.6. Raman Spectroscopic Imaging

The Raman spectra mainly revealed the localization of main compounds, leghaemoglobin, asparagine and glutamine, taking part in the nitrogen fixation process. Leghaemoglobin plays a fundamental role in nitrogen fixation by limiting oxygen partial pressure inside the nodule [30]. On the other hand, glutamine plays an essential role in the assimilation of the ammonium released by nitrogenase, and by serving as nitrogen donors for further biosynthesis of essentially all nitrogenous compounds. It can be directly exported from the nodules or used to synthetize asparagine, the main nitrogen export compound in indeterminate nodules [31].

**Figure 7:**
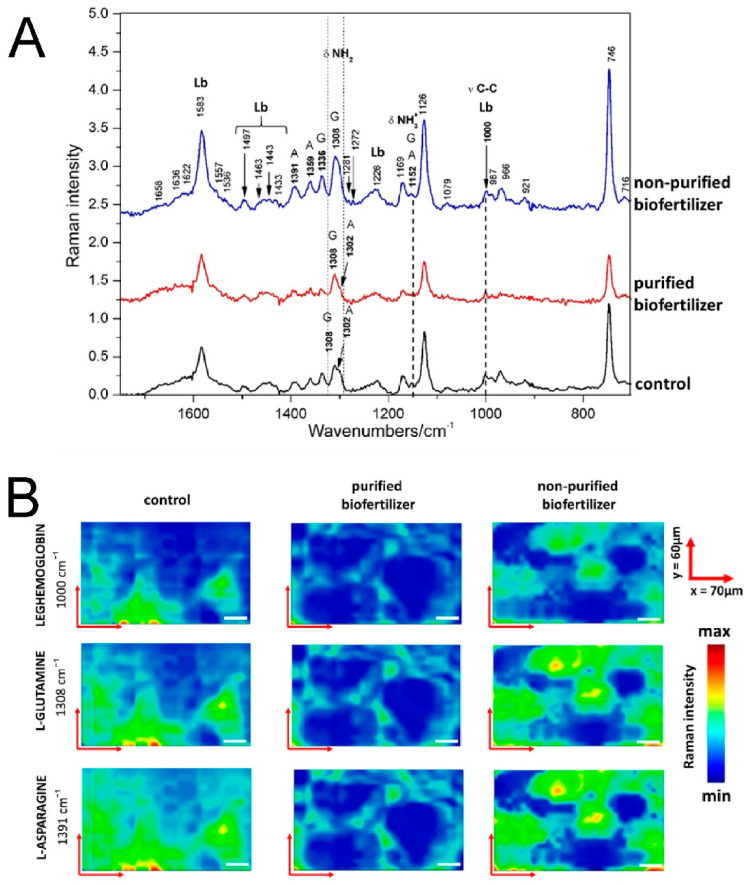
(**A**) Raman relative intensity spectra of the root nodules from plants grown from seeds soaked with water (control sample, black line), nodule from plant grown from seeds coated with purified biofertilizer (red line) and nodule from plant grown from seeds coated with non-purified biofertilizer (blue line). A—asparagine, G—glutamine, Lb—leghaemoglobin. Spectra are presented in the range of 1750–700 cm^−1^. (**B**) The Raman maps of the root nodules obtained for bands representing leghaemoglobin (1000 cm^−1^), glutamine (1308 cm^−1^) and asparagine (1391 cm^−1^) for control sample and root nodules from plants grown from seeds coated with purified and non-purified biofertilizer. Each map has the dimension 70 µm × 60 µm, white bar has 10 µm.

All average Raman spectra (Figure 7A) had bands characteristic for leghaemoglobin: C(in pyrrole group)-C(in vinyl group) symmetric stretching band at 1000 cm^−1^ and region 1400–1600 cm^−1^ responsible for porphyrin skeletal modes [32,33]. In the case of glutamine and asparagine, the characteristic bands in the region 1200–1400 cm^−1^ that could be assigned to the bending vibration of NH_2_, CH_2_, and CH groups, are present [33].

For further analysis of differences among samples, the bands: at 1000 cm^−1^ (C-C symmetric stretching band in leghaemoglobin), at 1308 cm^−1^ (NH_2_ in-plane scissoring coming from glutamine) and at 1391 cm^−1^ (CH_2_ in-plane scissoring coming from asparagine) were chosen (Figure 7A) [33,34]. The intensity of the C-C band symmetric stretching of leghaemoglobin had the highest intensity in the average spectrum of the control nodule. The lowest intensity was found in the spectrum of the nodules from plants grown from seeds coated with purified biofertilizer. The lower intensity of the C-C symmetric stretching band corresponded to the higher leghaemoglobin bonding of the NO_2_ group [33]. Taking this into consideration, it can be concluded that the nodule from plants treated with purified biofertilizer has the highest amount of NO_2_ bonded to leghaemoglobin; in the centre there is nodule from plants treated with non-purified fertilizer and the lowest amount of NO_2_ bonded to leghaemoglobin was found in the control nodule. The area of the band at 1308 cm^−1^ corresponding to NH_2_ in-plane scissoring of glutamine is the highest in the nodule from plants treated with non-purified fertilizer, while a lower intensity is presented by the spectra of the control nodule, and the lowest is found in the spectra of the nodule from plants treated with purified fertilizer. It should be noticed here that the band characteristic for asparagine at 1302 cm^−1^ found in the control nodule overlaps the band of glutamine and makes it wider. Thus, the height of the peak should be taken in consideration during the analysis of this band. The highest band is the one in the spectrum of the nodule from plants treated with non-purified biofertilizer. The bands of the nodules from plants treated with purified biofertilizer and the control sample have the same height. The area of the band at 1391 cm^−1^ corresponding to CH_2_ in-plane scissoring of asparagine is the highest in the nodule from plants treated with non-purified biofertilizer; it was lower in the nodule from plants treated with purified biofertilizer and was the lowest in the control sample nodule.

The changes visualized on the chemical maps are consistent with the single Raman spectrum (Figure 7A,B). The band corresponding to C-C symmetric stretching of leghaemoglobin at 1000 cm^−1^ is the most intense in the control sample; it is less intense in the nodule from plants grown from seeds treated with non-purified biofertilizer and has the lowest intensity in the nodule from plants grown from seeds treated with purified biofertilizer. The intensity is inversely proportional to the amount of fixed nitrogen. The band corresponding to NH_2_ in-plane scissoring of glutamine at 1308 cm^−1^ is the most intense in the nodule from plants treated with non-purified fertilizer, lower intensity in the control nodule and the lowest intensity in the nodule from plants treated with purified biofertilizer. The same results were obtained for the band at 1391 cm^−1^ corresponding to CH_2_ in-plane scissoring of asparagine (Figure 7B). The lower area (or height) of the bands (in the statistical spectra) and the lower intensity of the bands (on the chemical maps) corresponding to alkaline amino acids in the nodules form plants treated with purified biofertilizer, in comparison with nodules from plants treated with non-purified biofertilizer and in the control sample, might be due to the higher amount of the amino acid transporters that transport the amino acids from the nodules to plants in the sample in the nodule from plants treated with purified biofertilizer [35]. This might be partly explained by the higher amount of α-helix proteins (visualized and on deconvoluted Amide I band from the FT-IR spectroscopy) in the nodule from plants treated with purified biofertilizer, but this topic needs further investigation.

## 3. Materials and Methods

### 3.1. Reagents and Materials

2,5-Dihydroxybenzoic acid (DHB), trifluoroacetic acid (TFA) and naringenin were purchased from Sigma-Aldrich (St. Louis, MO, USA). Acetonitrile (ACN), iso-propanol (2-propanol, IPA), methanol (MeOH) and formic acid (FA), all in LC-MS grade, were provided by Merck (Darmstadt, Germany) and n-butanol was supplied by Avator Performance Materials (Gliwice, Poland). Pure water was produced on site using Milli-Q Elix system (Millipore Corporation, Bedford, MA, USA).

### 3.2. Isolation of Nod Factors

The bacterial strain *Rhizobium leguminosarum* bv. Viciae GR09 was grown for 48 h in twelve 1 L flasks containing 300 mL of TY medium, at 28 °C, with aeration by shaking (110 rpm, Orbitron, Infors HT, Bottmingen, Switzerland). The cultures, at a logarithmic phase of growth, were induced with naringenin (ethanol solution) at a final concentration of 10 mM, and the growth of cultures was continued under the same conditions for the next 48 h. Then, the bacteria were removed by centrifugation (9000 rpm, 15 min, 10 °C), and supernatants were collected and freeze-dried. The lyophilizate was dissolved in 200 mL of deionized water, placed into a separatory funnel and 40 mL n-butanol was added. The mixture was vigorously shaken for 10 min. Phases were separated at room temperature. The lower, butanol fraction was collected, and the aqueous phase was re-extracted with the addition of 20 mL n-butanol, and centrifuged (4000 rpm, 20 min, 20 °C). The butanol phases were pooled and evaporated using a vacuum rotary evaporator. The dry residue was dissolved in 50% acetonitrile and stored at −20 °C until used. The solution was divided in two parts—one was used as a non-purified biofertilizer, and the second part was subjected to purification by fractionation using the SPE column.

### 3.3. Fractionation of the Butanolic Extract Containing Nod Factors by SPE

A 20 mL volume column, containing 10 mL Supelclean^TM^ LC-18 SPE bed (Supelco, Bellefonte, PA, USA) was washed with 20 mL of deionized water, then a sample of 5 mL was flown. Subsequent steps included sequential washing with water, 50% aq. methanol, 60% aq. acetonitrile and 100% acetonitrile, each eluent used in 20 mL volume. The obtained fractions were evaporated using a vacuum evaporator and dissolved in 2 mL of solvent suitable for the given fraction. The preparations were stored at −20 °C. The method of production and fractionation of the Nod factor based biofertilizer was described in a patent application [P.433315].

### 3.4. UPLC and ESI-MS Analyses

Reversed phase chromatography was performed using Waters AQUITY Ultra Performance LC system (Milford, MA, USA), with an analytical column BEH C18 1.7 µm, (2.1 × 100 mm, beds diameter: 1.7 µm). The column was heated to 40 °C and eluted with a gradient of solvents from 99% A and 1% B to 1% A and 99% B, where: A: water, with 0.1% formic acid; B: acetonitrile, with 0.1% formic acid.

The flow rate of the mobile phase was 0.4 mL/min. The sample volume was 5–10 µL. Samples were dissolved in 50% acetonitrile and were injected using AQUITY autosampler.

ESI-MS spectrometry was performed with SYNAPT G2-S*i* HDMS instrument (Waters Corporation, Milford, MA, USA) operating in positive ion mode. Acquisition of the data were performed at a range of 100–2000 *m*/*z*, using MassLynx software, version 4.1 SCN916 (Waters Corporation, Wilmslow, UK). Mass spectra were assigned with a multi-point external calibration using sodium iodide (Sigma-Aldrich, St. Louis, MO, USA). Mass spectrometer conditions were as follows: capillary voltage: 3.00 kV, sampling cone: 40 V, source offset: 80 V. Ion source temperature was established at 100 °C and desolvation temperature: 200 °C. Cone gas flow was set at 100 L/h and desolvation gas flow—800 L/h.

### 3.5. Plant Experiments

Pea plants (*Pisum sativum* L. cv. Batuta) were grown in a greenhouse at the Faculty of Biology and Biotechnology of the Maria Curie-Sklodowska University in Lublin (Poland), in pots filled with a mixture of garden soil, sand and perlite (1:1:1; *v*/*v*/*v*). Pots were watered with water three times a week. Before sowing, the seeds were soaked for half an hour in three solutions: (1) non-purified biofertilizer (2) purified biofertilizer fraction 60% acetonitrile (both preparations were diluted 1000 times with water) and (3) control—seeds soaked with water. Each pot contained 6 seeds. Each experiment was repeated twice.

After 21 and 42 days of cultivation, two randomly chosen pots from each of the experimental groups were taken, the root nodules were counted, the fresh mass of shoot and root were weighted, and then the material was dried in order to determine the weight of shoot and root dry masses. Statistical analyses were carried out by the analysis of variance and Tukey test using the STATISTICA software (ver. 13.3, Stat Soft, Tulsa, OK, USA).

A set of root nodule thin slices was prepared for each imaging spectroscopic technique. 

### 3.6. MALDI-MSI Sample Preparation

Samples of root nodules collected from plants grown for 21 and 42 days were trimmed away from the plant and individually flash-frozen in liquid nitrogen for 10 s. The frozen nodules were longitudinally sectioned into 30 µm slices using cryomicrotome at −20 °C (Leica CM 1950, Leica Biosystems, Wetzlar, Germany). The sections were placed on a standard no frost glass microscope slides (Thermo Fisher Scientific, Waltham, MA, USA). Four root sections were prepared for MALDI MSI optimization and three sections from each plant were used for the analysis. Then, the MALDI matrix (2,5-dihydroxybenzoic acid (DHB), 40 mg/mL in 50% aq. methanol + 0.1% TFA) was applied on the tissue using airbrush.

### 3.7. MALDI MSI Data Acquisition

MALDI mass spectrometry imaging analyses were performed in positive ion mode using MALDI-TOF instrument (Synapt G2-S*i*, Waters Corporation, Milford, MA, USA), equipped with Nd:YAG laser system. The laser power was optimized and set up at 250 eV with a reception rate of 1000 Hz. The laser step size was 60 µm × 60 µm (in (*x*, *y*) axis). The measurement was performed in 100–1500 *m*/*z* range. MS imagining data were analysed using HDImaging (ver. 4.1, Waters Corporation, Wilmslow, UK). Selected metabolites were identified by trawling the accurate masses obtained by the mass spectrometer. The masses and possible adducts such as [M + H]^+^, [M + Na]^+^, [M + K]^+^, were searched for, using ChemSpider and PubChem databases.

### 3.8. Sample Preparation for FTIR and Raman Imaging

Samples of root nodules collected from plants grown for 21 and 42 days were trimmed away from the plant and individually flash-frozen in liquid nitrogen for 10 s. The frozen nodules were longitudinally sectioned into 30 µm slices using cryomicrotome at −20 °C (Leica CM 1950, Leica Biosystems, Wetzlar, Germany). The sections were placed on aluminium coated microscope slides.

### 3.9. FT-IR ATR Spectra Acquisition

In order to perform semi-quantitative FT-IR analysis at least nine nodules were collected from each group: control, non-purified biofertilizer and purified biofertilizer, respectively. They were desiccated, pressed to create a thin film, and measured with the diamond ATR crystal with DTSG KBr detector using Nicolet 6700, (Thermo Scientific, Waltham, MA, USA). The spectra were obtained in the 4000–400 cm^−1^ range, with a spectral resolution of 4 cm^−1^. Each spectrum resulted from 32 measurements. Spectra were normalized to the band at 2925 cm^−1^. The spectra presented on Figure 6 were averaged over nine spectra (one from each nodule). The deconvolution of the averaged spectra was performed in respect to the second derivative spectra using Origin Pro Software (v. 9.1, OriginLab Corporation, Northampton, MA, USA). Moreover, each spectrum obtained by the use of the ATR-mode was deconvoluted in order to perform the statistical analysis of the area under selected bands. The spectrum with the highest differences from the average one was excluded from the statistical analysis. The statistical differences were shown using HSD-Tukey test (post hoc test, comparing all possible pairs of means, based on studentized range distribution, similar to *t*-test).

### 3.10. FT-IR Spectroscopic Imaging Data Acquisition

FT-IR spectra were collected in transflection mode using Nicolet 6700 FT-IR spectrometer (Thermo Scientific, Waltham, MA, USA). Spectra were recorded in the spectral range of 4000–650 cm^−1^ with a spectral resolution of 8 cm^−1^. Each spectrum resulted from 120 scans to obtain an optimal signal-to-noise ratio. The aperture used was 100 µm diameter. The magnification of the objective was 15×. Maps were obtained with the step size 150/150 µm in the *x* and *y* axis. Baseline corrections and further analysis of the maps were performed using CytoSpec (v. 2.00.01).

### 3.11. Raman Spectroscopic Imaging Data Acquisition

Raman spectra were collected using DXR Raman confocal microscope (Thermo Scientific, Waltham, MA, USA) with semi-conductive laser emitting λ = 532 nm wavelength with maximum power at 10 mW. Spectra were recorded in the spectral range of 3750–250 cm^−1^ with a spectral resolution of 4 cm^−1^. Each spectrum resulted from 6 scans to obtain an optimal signal-to-noise ratio. The aperture used was 50 µm diameter. Raman chemical maps were obtained with the step size 2/2 µm in the *x* and *y* axis. The single spectra were averaged over 120 spectra from the root nodules and normalized to the lipid band at 2936 cm^−1^. Baseline corrections and further analysis of the spectra were performed using Omnic Software (v. 8.2, Thermo Fischer Scientific, Waltham, MA, USA).

## 4. Conclusions

Within this study, it was demonstrated that both purified and non-purified biofertilizers stimulate the growth and development of pea plants; however, the purified preparation is more effective. In order to determine the factors involved in the action of the biofertilizer, spectroscopic analyses were performed using MALDI-MSI, FT-IR imaging and Raman imaging. These enabled visualization of the changes in the amount and spatial location of the nodule metabolites.

Purified biofertilizer increased the amount of α-helical proteins and also the amount of leghaemoglobin. Interestingly, purified biofertilizer decreased the amount of basic amino acids in the nodule, which might be due to the higher amount or efficiency of the basic amino acid transporters. Moreover, it induced accelerated nodule maturation and abridgement of the lipids.

Non-purified biofertilizer apparently caused an increase in the protein content, but mainly the amount of β-sheet structure proteins, thus the amount of leghaemoglobin presumably increased, although only slightly. The lower amount of α-helical proteins also correlated with the higher amount of basic amino acids, possibly due to the higher efficiency of nitrogen fixation than the control samples, but lower efficiency of the nitrogen species transport (lower amount of basic amino acids transporters). Non-purified biofertilizer also did not accelerate the maturation of the nodules. Moreover, it favoured longer lipid chains.

Obtained results confirm the usefulness of Nod factor-based biofertilizer in the stimulation of pea growth, and it can be assumed that such biofertilizers can be a good alternative for the application of synthetic nitrogen fertilizers.

In addition, we can state that this study demonstrated that different chemical imaging spectroscopic methods can be used as effective tools to study the molecular distribution of nitrogen fixation metabolites. These methods can enable investigations into environmental and human influences on organisms.

## 5. Patents

This paper has taken part in the patent application [P.433315] (https://ewyszukiwarka.pue.uprp.gov.pl/search/pwp-details/P.433315?lng=en (accessed 27 November 2021)).

## Figures and Tables

**Table 1:** Identification of Nod factors in the preparation obtained from *R. leguminosarum* bv. Viciae strain GR09, fraction eluted with 60% acetonitrile.

Signal	Observed Signal [M + H]^+^	Calculated Molecular Mas [M + H]^+^	Assignment
A	889.4956	889.4783	3 × GlcN; 18:3; Ac
B	1002.5784	1002.5380	3 × GlcN; 20:2; 3 × Ac
C	1083.5635	1083.6170	4 × GlcN; 20:0
D	1322.7389	1322.6600	5 × GlcN; 20:3; Ac

Ac—O-acetyl group.

## Data Availability

The data that support the findings of this study are available from the corresponding author upon reasonable request.
